# Repeated computed tomography scanning reveals morphological development of burrows produced by the tiger pistol shrimp *Alpheus bellulus*

**DOI:** 10.1371/journal.pone.0273055

**Published:** 2022-08-29

**Authors:** Miyu Umehara, Koji Seike, Seishiro Furuyama

**Affiliations:** 1 Tokyo University of Marine Science and Technology, Tokyo, Japan; 2 Geological Survey of Japan, National Institute of Advanced Industrial Science and Technology (AIST), Tsukuba, Ibaraki, Japan; 3 Department of Natural Environmental Studies, Graduate School of Frontier Sciences, The University of Tokyo, Kashiwa, Chiba, Japan; University of Liverpool, UNITED KINGDOM

## Abstract

The burrow morphology of endobenthic organisms reflects their subsurface ecology. In this study, we observed the three-dimensional development of burrows produced by the tiger pistol shrimp *Alpheus bellulus* in a tank using an X-ray computed tomography (CT) scanner. CT scanning was performed at 10–30 min intervals immediately after the start of burrow construction. The three-dimensional morphology (surface area, volume, depth, length, and diameter) of burrows at each observation time was imaged and measured. In addition, the rate of increase of each parameter was calculated. Surface area, volume, length, and depth rapidly increased immediately after the start of the experiment in all burrows. Subsequently, there was a reduction in the rate of increase at 40 min after the start of excavation for burrow depth, at 75 min for length, and at 90 min for surface area and volume. Although there were large differences in burrow diameter among the burrows immediately after the start of the experiment, all burrows reached nearly identical diameters after 90 min. Changes in burrow morphology were not observed in most of the burrows more than 210 min after the start of the experiment, meaning that *A*. *bellulus* can create burrows that are sufficient for survival within this time period. The use of CT scans in this study clarified the developmental process of the three-dimensional structure of *A*. *bellulus* burrows and is applicable to various burrow-producing organisms. Our results provide new insights into the development of burrow structures.

## 1. Introduction

Burrows in marine environments have existed throughout the world since the Cambrian explosion when benthic organisms started to make a burrow acting as a refuge from predators [1], and have also been reported from modern and ancient extreme environments such as deep-sea hydrocarbon seeps [2, 3]. The ecology of these organisms can provide insights into marine ecosystems from past to present. However, because burrowing benthic organisms are often buried in sediments, it is difficult to observe their behaviors *in situ*. Therefore, much of their subsurface ecology remains unknown.

The burrows of benthic organisms are generally characteristic, and can be distinguished by taxon or feeding ecology [4]. Although the development of burrows has been observed using thin experimental tanks [5, 6], this method only provides two-dimensional information on burrow structure as viewed from the side of the tank. Resin casting of burrows in experimental tanks (mesocosms) has also been used to observe three-dimensional burrow structures [7]. Although casting can provide useful information even on burrows produced by large crustaceans such as alpheid shrimps, the method is inherently destructive and can only be used to identify the burrow morphology at a moment in time, making it unsuitable for investigating the process of burrow construction. Sediment mixing by benthos through the burrow construction is one of the most important controls on biogeochemical cycling and vertical sediment redistribution in seafloor ecosystems [8]. Revealing dynamics of burrow construction is thus important if we are to understand better the interaction between benthos and benthic marine environments.

**Fig 1:**
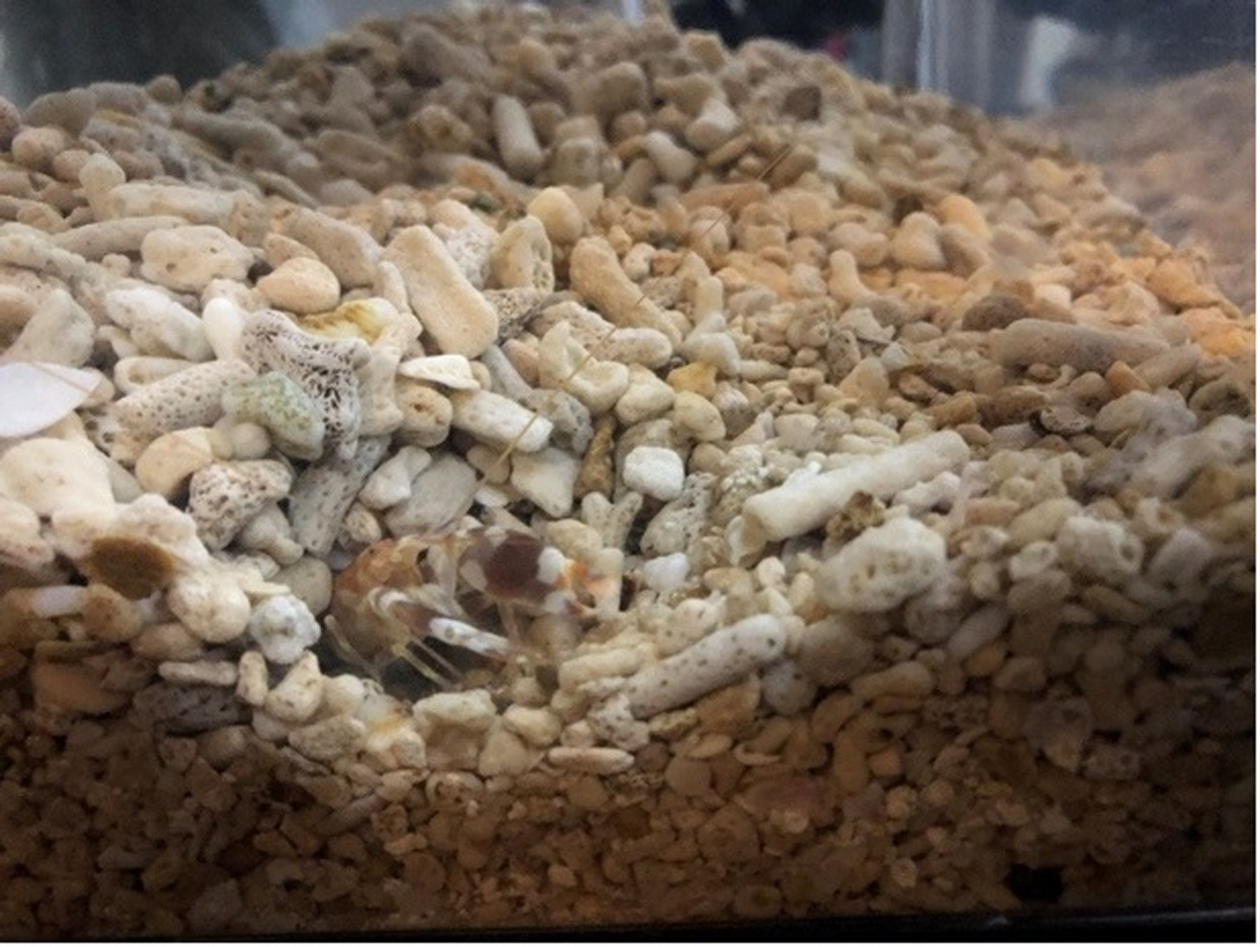
The tiger shrimp *Alpheus bellulus* near a burrow entrance in an experimental tank.

Minter et al. [9] conducted repeated micro-X-ray computed tomography (CT) scans to observe the four-dimensional morphology (i.e., the time dimension plus three spatial dimensions) of ant burrows. This method has since been applied to observe the development of other insect nests such as those belonging to social bees [10], and is also applicable to burrows of marine benthic organisms. In this study, repeated X-ray CT scanning was performed on the tiger pistol shrimp *Alpheus bellulus* (Crustacea, Decapoda, Alpheidae) in experimental aquaria (Fig 1) to clarify the development of burrows in this species. Alpheus shrimps are representative large body-size burrower in tropical and temperate shallow marine settings throughout the world, suggesting the shrimp is an ideal benthic organism to study burrowing activity as a model system. To our knowledge, ours is the first study to apply repeated X-ray CT scanning to marine benthos, which enabled us to make accurate observations of burrow morphology in four dimensions. We measured carapace length of the shrimp and compared it with burrow parameters, because the burrowing capabilities of benthic organisms are known to be affected by their body size [11, 12]. Our method could be used to provide valuable insights into the ecology of marine burrowing animals more generally.

## 2. Materials and methods

### 2.1. Study species

We observed five *A*. *bellulus* (Crustacea, Decapoda, Alpheidae) individuals in the laboratory for this study. These individuals were sourced from an aquarium shop in Tsuchiura, Ibaraki Prefecture, Japan. This species is widely distributed in the Indo-Western Pacific, and its congeners are common in tropical and temperate shallow marine settings throughout the world. Of the more than 300 *Alpheus* species described so far [13], about 60 have been reported from Japan. *Alpheus bellulus* has been identified in Japan from the Izu Peninsula [14], Kii Peninsula [15], Okinawa Islands [16], and Yaeyama Islands [17]. The shrimp constructs subtidal burrows in sediments that are typically a mix of sand, pebbles, coral debris, and shell fragments. In the field, *A*. *bellulus* burrows reach 30–70 cm in length and 70 cm in depth [15]. Burrows are known to harbor commensals such as the gobiid fishes *Amblyeleotris japonica*, *Amblyeleotris diagonalis* [17], *Tomiyamicthys oni* [15], and *Stonogobiops pentafaciata* [18].

### 2.2. Experimental setup

**Fig 2:**
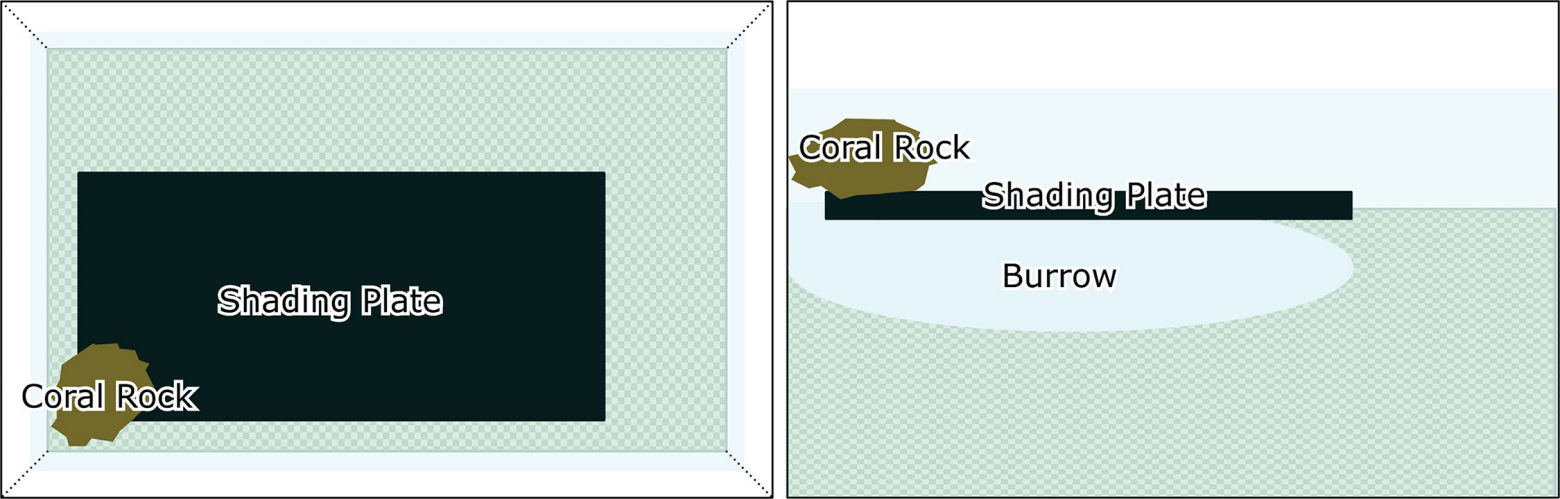
Schematic diagram of an experimental tank. The left and right panels show top and side views, respectively.

The five study animals were identified as individual A (IA) through E (IE). Their carapace lengths were 13.6 mm, 19.4 mm, 13.3 mm, 17.4 mm, and 15.8 mm, respectively. Prior to experiments, all shrimps were maintained in the laboratory under the same constant environmental conditions as those of the experiment for over 10 days to ensure that any endogenous or physiological rhythms were abolished. IA and IB were first housed in their experimental tanks on 22 July 2020, and IC, ID, and IE were first housed on 17 September 2020. Each shrimp was placed in a separate plastic tank (dimensions, 41 cm × 24 cm × 15.5 cm; Fig 2). The tops of the tanks were covered with plastic wrap to prevent the shrimp from escaping, and the tanks were filled with fragmented coral sand up to a depth of around 7 cm. In addition, a plastic plate (14 cm × 30 cm; Fig 2) was placed on the sand layer to provide shade and promote burrowing, because the congeneric *A*. *djiboutensis* has been reported to show negative phototaxis [19]. Also, this plate helped the experimental animals establish and reinforce their burrow openings; in the field, they generally use shells and coral rocks for this purpose. A coral rock was placed at one corner of each tank to fix the location where the shrimp would construct a burrow (Fig 2). Each shrimp was placed near the coral rock to induce them to burrow at one corner of each tank. Water temperatures were maintained at approximately 25°C, and salinities at approximately 34. Shrimps were fed granular marine fish food to satiation, three times a week. The shrimp did not appear to respond to the provided food in the early stages of burrow construction. This study was carried out in strict accordance with the recommendations in the Guide for the Care and Use of Laboratory Animals of the National Institutes of Health.

### 2.3. X-ray CT scans

After acclimatization to constant environmental conditions for 10 days or more, tanks with active burrows were subjected to X-ray CT scanning (Supria Grande Premium, Hitachi, Ltd., Japan; installed at the Geological Survey of Japan, National Institute of Advanced Industrial Science and Technology) at power settings of 80 kV and 250 mA and a slice thickness of 0.625 mm. The pixel area was 0.59 mm^2^, and the voxel volume was 0.369 mm^3^. Each tank was scanned at 10-min intervals during the first hour after the initiation of burrowing, then at 15-min intervals for the next half-hour, and finally at 30-min intervals for the subsequent 3 h (i.e., at 0, 10, 20, 30, 40, 50, 60, 75, 90, 120, 150, 180, 210, 240, and 270 min after the start of burrowing). No negative effects of the repeated X-ray scanning on shrimp burrowing activity were observed during the experiment; burrowing activity by the shrimp after the end of the experiment appeared to be the same as the activity observed prior to the experiment.

### 2.4. Data analysis

**Fig 3:**
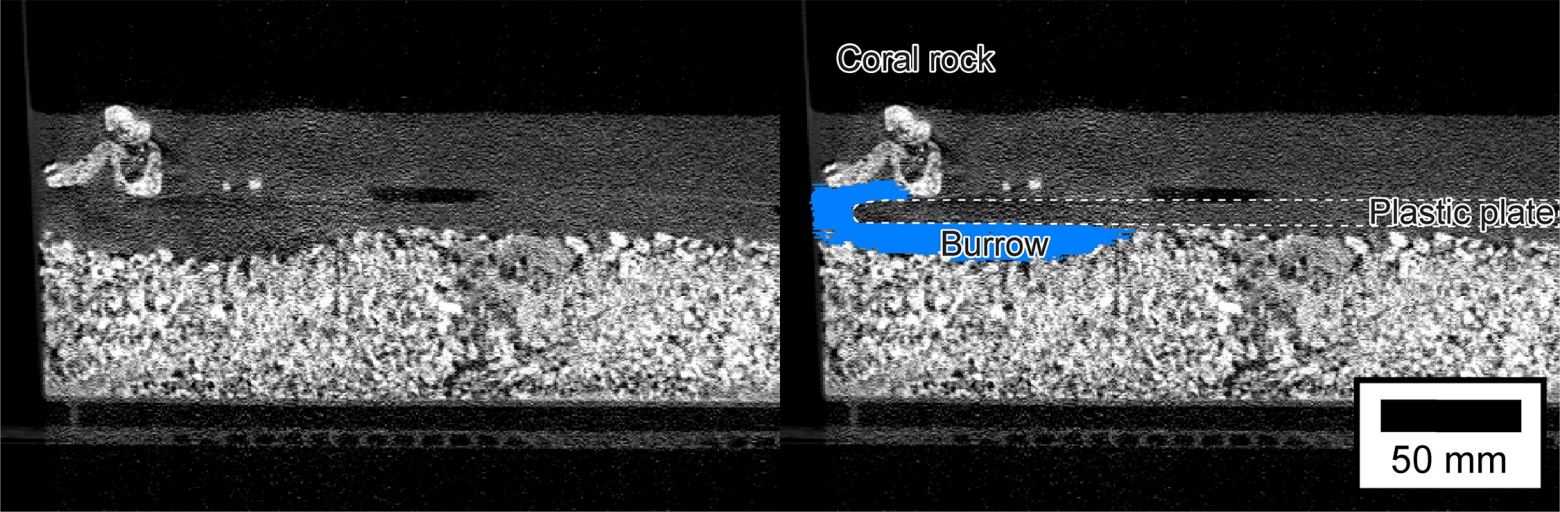
Cross section of an *Alpheus bellulus* burrow imaged by X-ray computed tomography. The blue shaded region in the right-hand panel shows the location of the burrow.

**Fig 4:**
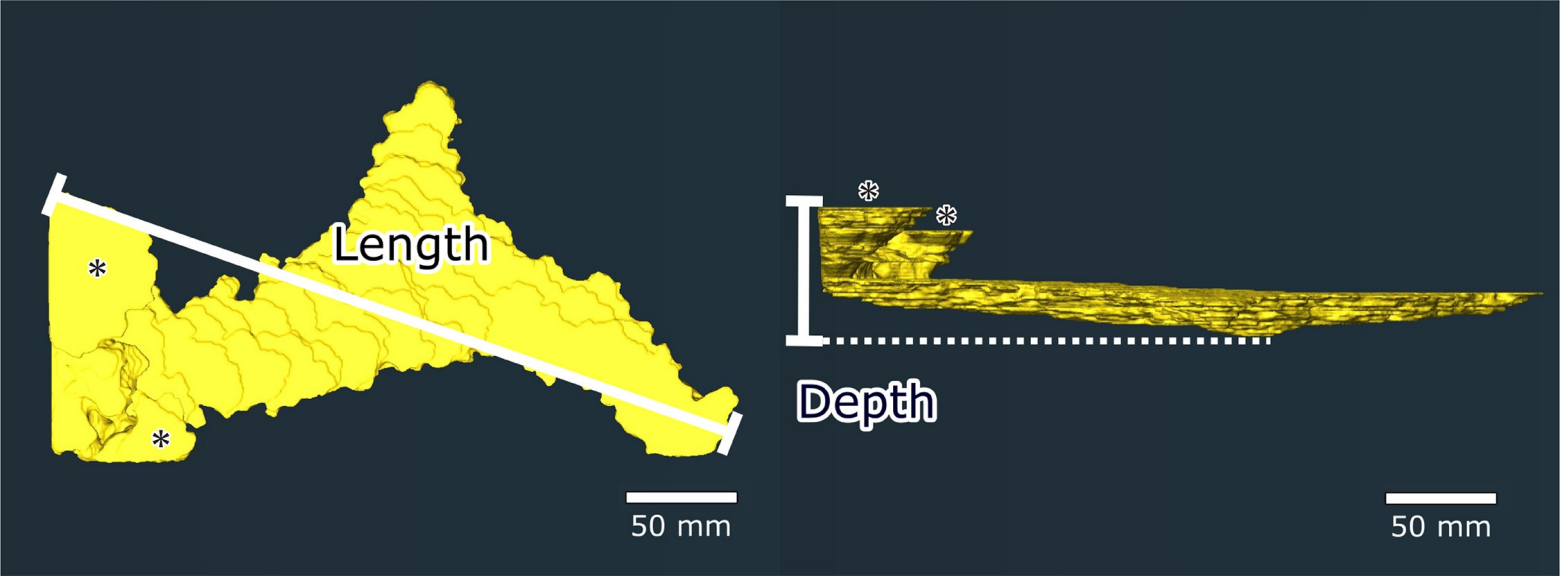
Measurements of burrow length and depth from computed tomography images. The left-hand and right-hand panels show top and side views, respectively. The asterisks show the burrow apertures.

The Amira 2019.2 software platform with X Image PAQ Extension (Thermo Fisher Scientific, Ltd., USA) was used to analyze three-dimensional images obtained by the X-ray CT scans. The cross-sectional (two-dimensional) shape of the burrows in each X-ray image were traced to obtain three-dimensional images of the burrows at each scanning timepoint (Fig 3). The surface area, volume, depth, and diameter of the burrows (Fig 4) were computed from 3D image data using the Surface Area Volume, Thickness Map, and Label Analysis modules. Burrow diameter was defined as a modal value of local thickness at each point inside the burrow [20]. Burrow length was defined as the maximum horizontal extent of a burrow, and was measured using Adobe Illustrator (Fig 4). Maximum rates of increase (i.e., the magnitude of change per minute) of each burrow parameter were calculated by comparing the burrow parameter data among scanning timepoints. We used statistical software R 4.1.2 to conduct Pearson’s correlation tests to determine whether shrimp carapace length and burrow parameters were correlated. The null hypothesis was that there was no association between the two selected variables; this was rejected if the *p*-value did not exceed 0.05.

## 3. Results

### 3.1. Morphodynamics of shrimp burrows

**Fig 5:**
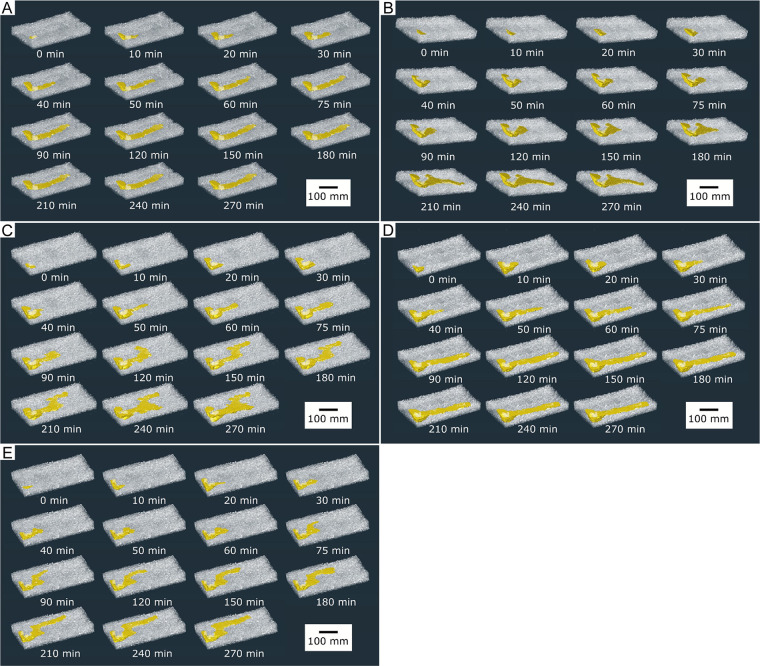
Changes in burrow morphology over time. Panels show the following: (A) Individual A, (B) Individual B, (C) Individual C, (D) Individual D, and (E) Individual E.

X-ray CT images showed the three-dimensional shape of the burrows constructed by the five individual *A*. *bellulus* shrimp (Fig 5). All five shrimp constructed their burrows under the plastic plates provided for shading, and all burrow openings were located near the coral rocks. The burrows were horizontally elongated in shape, and could be classified into two morphological types: straight unbranched (produced by IA and ID) and branched/bent (produced by IB, IC, and IE). The maximum dimensions of the five burrows ranged as follows: surface area, 44,875–68,398 mm^2^; volume, 155,005–255,850 mm^3^; depth, 44–55 mm; and length, 309–357 mm.

**Fig 6:**
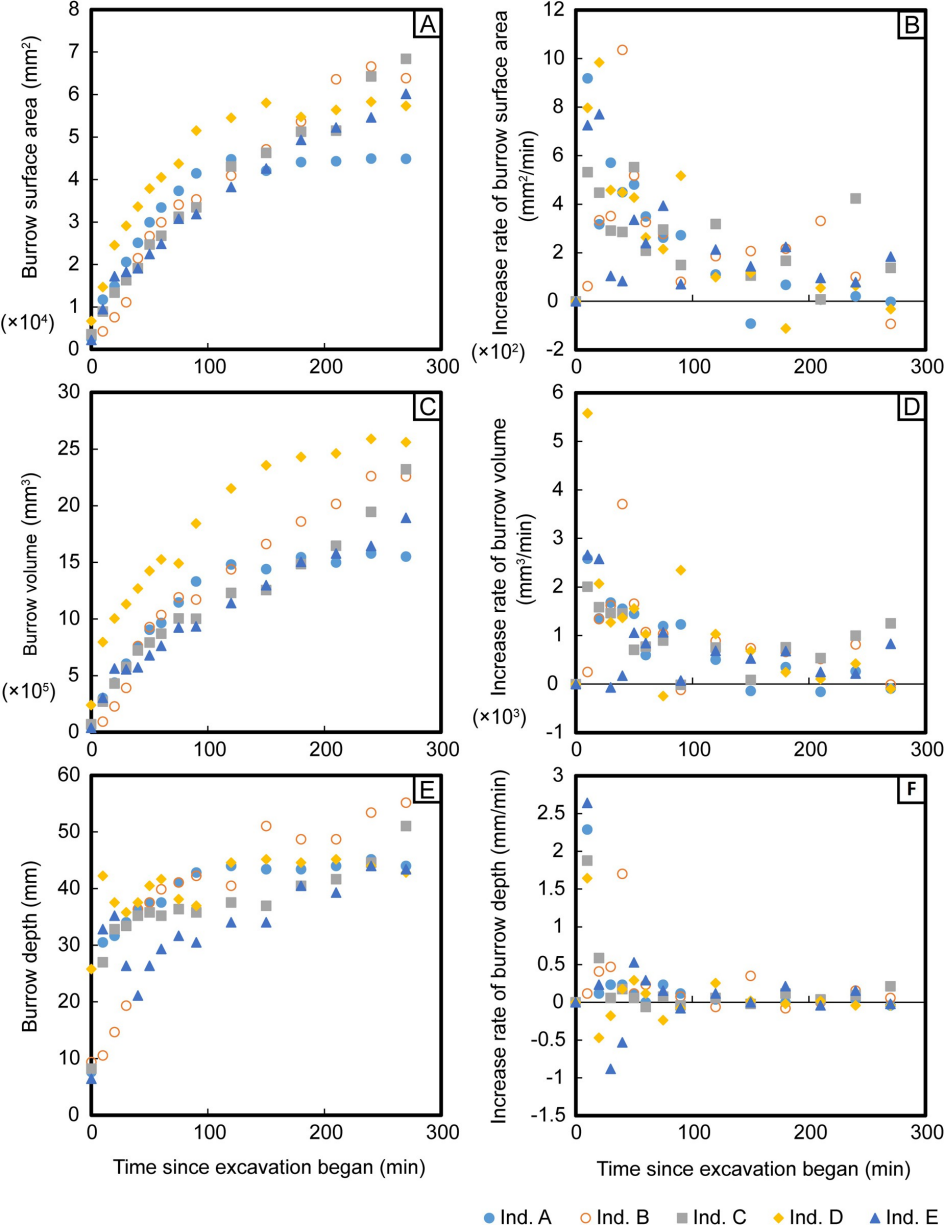
Changes in burrow surface area, volume, and depth over time. Panels show: (A) surface area, (B) the rate of increase of surface area, (C) volume, (D) the rate of increase of volume, (E) depth, and (F) the rate of increase of depth.

**Fig 7:**
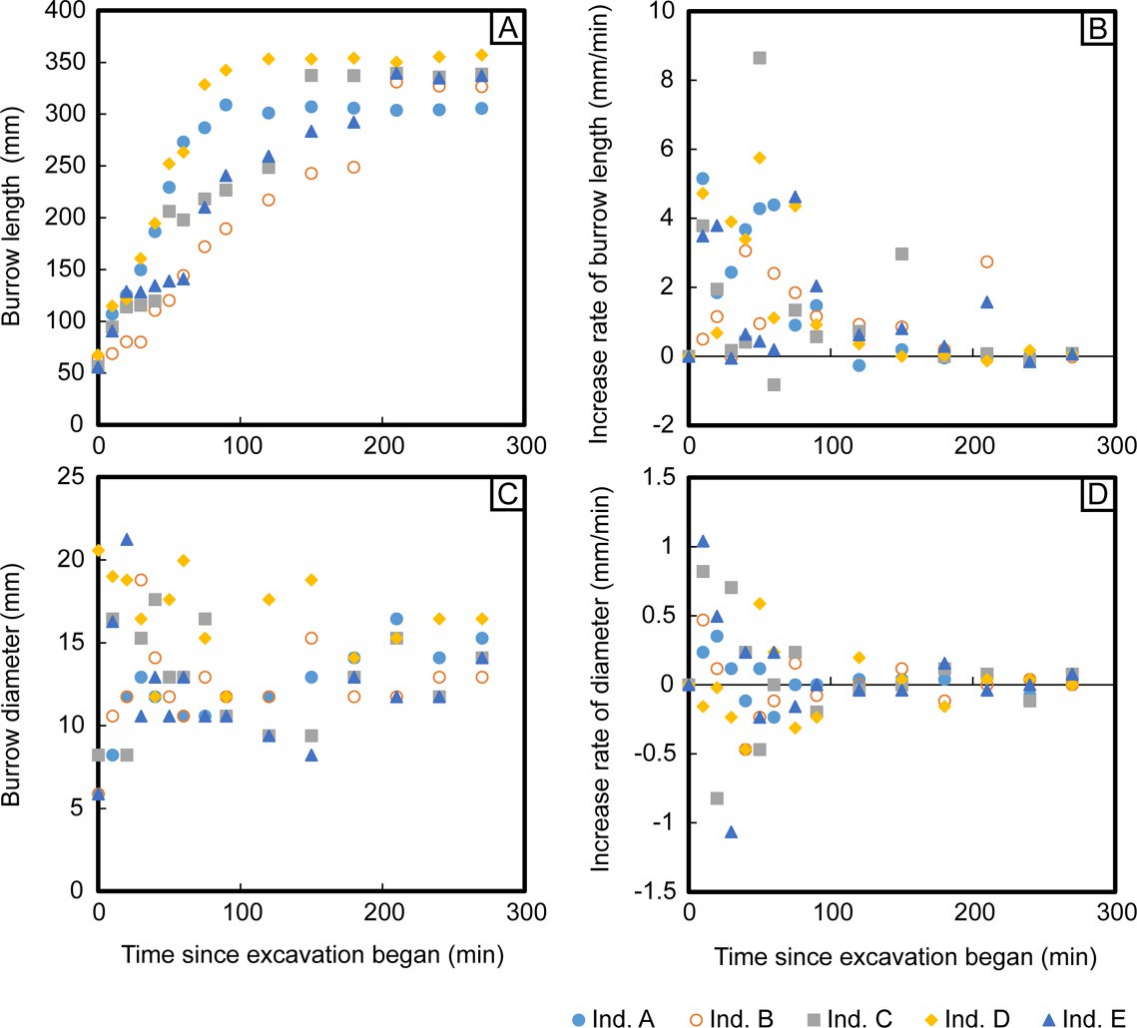
Changes in burrow length and diameter over time. Panels show: (A) length, (B) the rate of increase of length, (C) diameter, and (D) the rate of increase of diameter.

The surface areas and volumes of most of the burrows began to increase immediately after the start of the experiment (Fig 6A and 6C). Although all parameters except for burrow diameter continued to increase throughout the duration of the experiment, the rates of increase declined beginning at 90 min since the start of excavation, and the maximum rates of increase occurred somewhere between the start of the experiment and 40 min since the start of excavation (Fig 6B, 6D and 6F). Although burrow diameters differed among burrows at the beginning of the experiment, they converged over time to a narrow range of 12.9–16.4 mm (Fig 7C and 7D). Burrow depths increased rapidly at the start of the excavation and then continued to increase more gradually (Fig 6E and 6F). At the end of the experiment, some burrows were still increasing in depth, while others had leveled off (Fig 6E and 6F). Burrow length increased immediately after the start of the excavation in most burrows, and the rate of increase declined gradually over time, reaching zero for all burrows by the end of the experiment (Fig 7A and 7B).

The progression of burrow morphology for each shrimp was as follows:

IA constructed a straight unbranched burrow. All burrow parameters increased at their maximum rate immediately after the start of the excavation. Both burrow surface area and volume increased as a function of burrow length until the burrow length reached its maximum extent at 90 min. Burrow surface area and volume then continued to increase as a function of burrow depth until 120 min. At the end of the experiment, the expansion of the burrow had stopped.

IB constructed a branched burrow. All burrow parameters increased gradually immediately after the start of the excavation, and the rates of increase reached their maxima at between 40 to 50 min. After 75 to 90 min, burrow surface area and burrow volume both leveled off until the burrow developed a branch at 150 min, after which both parameters once again began to increase. Burrow length and surface area increased sharply after 180–210 min due to a substantial elongation of the branch. At the end of the experiment, the expansion of the burrow had stopped.

IC constructed a branched burrow. The rates of increase of burrow volume and depth were highest immediately after the start of the excavation. The rate of increase of burrow surface area peaked at 40–50 min, and increased again 210–240 min after the development of a branch. At the end of the experiment, burrow depth, burrow surface area, and burrow volume were continuing to increase.

ID constructed a straight unbranched burrow. The rates of increase of burrow volume and depth were highest immediately after the start of the excavation. Increases in burrow length stopped almost completely after 90 min, but burrow depth increased considerably at 90–120 min. At the end of the experiment, the expansion of the burrow had stopped.

IE constructed a bent burrow. The rates of increase of burrow surface area, volume, and depth were highest immediately after the start of the excavation. The rate of increase of burrow length reached an initial peak immediately after the start of the experiment, and a second peak at 60–75 min, when the bent portion of the burrow was formed. Burrow length and depth were no longer increasing by the end of the experiment, but burrow surface area and volume continued to increase due to increases in burrow diameter.

### 3.2. Relationships between shrimp carapace length and burrow parameters

The relationship between carapace length and the maximum value of each burrow parameter was as follows: There was a positive, though not statistically significant, correlation between carapace length and the rates of increase of burrow surface area (*n* = 5, *r* = 0.740, *p* = 0.152; Pearson’s correlation) and volume (*n* = 5, *r* = 0.717, *p* = 0.173; Pearson’s correlation). However, there was almost no correlation between carapace length and burrow surface area (*n* = 5, *r* = 0.339, *p* = 0.577; Pearson’s correlation), volume (*n* = 5, *r* = 0.502, *p* = 0.389; Pearson’s correlation), depth (*n* = 5, *r* = 0.375, *p* = 0.536; Pearson’s correlation), length (*n* = 5, *r* = 0.406, *p* = 0.497; Pearson’s correlation), or diameter (*n* = 5, *r* = 0.094, *p* = 0. 864; Pearson’s correlation).

## 4. Discussion

### 4.1. Burrowing activity

Most of the burrows reached their highest rates of increase of surface area, volume, depth, and length immediately after the start of the excavation, suggesting that the shrimp invest maximum effort initially to create their burrows. As described in section 2–2, the shrimp did not respond to feeding during this early stage, suggesting that they prioritized burrowing over foraging and resting. Similar behaviors have been reported for *A*. *macellarius* and *A*. *glaber* [21–23], indicating that the high initial priority assigned to burrowing is common among alpheids. This behavior is likely to help the shrimp escape from predation. In a natural setting, alpheid shrimp burrow often harbors commensal non-burrowing gobiid fishes, which act as alarm system against predators [21]. Alpheid shrimps, especially individuals that have temporarily lost their associated fish partner, need to construct their burrows quickly because their poor vision and reliance on tactile communication alone make them susceptible to predation in the field [21]. In our experiment, the shrimp were therefore essentially defenseless initially, and needed to burrow into the sediment to hide from predators as soon as possible.

### 4.2. Burrow morphology

The shapes of *A*. *bellulus* burrows have been previously identified through the use of burrow casting [15]. In the field, burrows typically have a funnel-shaped opening with a branch extending into an underground chamber. This is similar to the burrow openings observed in our study. Also, the subsurface chambers of burrows in the field are generally constructed under hard objects such as coral boulders or sand dollar tests [15]. In our study, the subsurface chambers were constructed under the plastic plates provided for shading, which may be analogous to the hard objects used in the field. One difference between the burrow morphology observed in our study and those described in the field is the shape of the burrow branches: the burrows in our study did not contain long branches extending into the chamber, and the burrow openings connected directly to the chamber. Yanagisawa [15] suggested that the overall morphology of burrows might be determined by the distribution of hard boulder materials such as those used to form the chamber. In this study, the shading plate might have acted as the hard object, thereby allowing the shrimp to form their chambers directly underneath it. Also, the size of the tanks could have limited the space available for long branches.

### 4.3. Relationships between shrimp carapace length and burrow parameters

Shrimp carapace length showed a positive, though not statistically significant, correlations with the rates of increase of burrow surface area and volume. This implies that larger shrimp were able to excavate sediments more quickly.

Henmi et al. [7] made resin casts from the burrows of *A*. *brevicristatus* in a mesocosm and found positive correlations between carapace length and burrow volume, depth, length, and diameter. Indeed, individuals with larger carapaces would be expected to construct larger burrows to accommodate their increased body size. However, our results do not support a positive correlation between carapace length and maximum values of burrow surface area, volume, depth, length, or diameter. The range of carapace lengths in our study (13.3–19.4 mm) might have been sufficient to see an effect of carapace length on burrow morphology because range width of carapace lengths in Henmi et al [7] was similar (6.16–11.71 mm), This lack of correlation can be attributed to several possible factors.

One possible factor is the limited time and space provided for burrow development in our study. Henmi et al. [7] measured burrow depths three weeks after the start of their experiment, ensuring that burrows were observed only once they were fully developed. In our study, the deepest burrow depth was measured at the end of the experiment (i.e., 4.5 h after the start of burrowing, in the burrow constructed by IC), when burrow depth was still increasing. Additionally, this burrow depth (about 55 mm) was not far off the 70-mm thickness of the sediment used in our study. Longer observation periods and larger tanks with thicker sediment layers should be used in future studies to allow the shrimp to construct more fully-developed burrows.

Another possible factor is the size of the shading plate used in our experiment. All shrimp in our study placed their burrow openings near the coral rock and constructed their burrows exclusively under the shading plate. Considering the diagonal length of the plate (about 330 mm) and the diameter of the coral rock (about 30 mm), the maximum horizontal length of any burrow that satisfies these conditions would be about 360 mm (Fig 2). This burrow length was reached by shrimp IE just 120 min after the start of the experiment, indicating that the burrow length was likely limited by size of the plate.

Although we did not find any correlation between carapace length and burrow diameter in our study, significant positive correlations have been reported for the congeneric *A*. *brevicristatus* [7]. This is likely because of the difference in burrow structure between the two species. Burrows constructed by *A*. *brevicristatus* have a branch-like structure in which the burrow diameter matches the size of the body [7]. However, the *A*. *bellulus* burrows observed in our study did not have a branch-like structure, and the bulk of the burrow consisted of the chamber dug under the shading plate. The diameter of this chamber did not appear to be correlated to carapace length. In addition, the lack of correlation can be attributed to the small sample size (*n* = 5).

### 4.4. Benefits of X-ray CT scanning

Minter et al. [9] used micro-CT scanning to observe the process of burrow development in the ant *Lasius flavus* and found that exogenous environmental features such as sediment boundaries affect nest morphology. The advantage of this method is that it allows researchers to nondestructively observe temporal changes in nest internal structures in three dimensions [24]. However, a limitation of micro-CT scanning is it can only image small samples. In contrast, we used a standard medical X-ray CT scanner in this study, which can take images of up to 500 mm in width and 1500 mm in length. The obtained images allowed us to visually and quantitatively analyze the process of burrow construction over time. This method is especially promising for large-bodied burrowing organisms. Our visual analysis allowed us to categorize the burrow morphologies into two types: straight unbranched and branched/bent. Our quantitative analysis revealed variations in burrow parameters at different stages of burrow development. Experiments using larger tanks could be conducted to make similar observations of even larger benthic organisms. Medical X-ray CT scanners are widely available due to their use in hospitals, and could prove valuable for a wide range of benthic organism research.

Although repeated exposure to X-ray radiation could conceivably alter the behavior of experimental animals, this is unlikely to have affected our results. Minter et al. [9] reported that excavation activity of the ant *L*. *flavus* was not affected by exposure to X-ray CT scanning, and in the present study, the *A*. *bellulus* individuals used in our research were still constructing burrows more than a year after our X-ray CT experiments (K Seike, personal observation). This suggests that the levels of radiation used in our experiment will not affect the survival and behavior of other large crustaceans, including other *Alpheus* shrimps, but more data is needed to confirm this. CT scanning of burrows will likely prove to be a highly useful and versatile method for quickly obtaining quantitative four-dimensional data on burrow morphology.
